# Anxiety and Depression Symptoms in Patients with Burn Injuries at Fundación Santa Fe de Bogotá, Colombia: Study of Patient-Reported Outcomes

**DOI:** 10.3390/ebj7030044

**Published:** 2026-08-13

**Authors:** Tatiana De Castro-Botero, Carlos Kerguelen-Botero, Natalia Botero-Tovar, Viviana Gómez-Ortega, Juan Diego Castro-Córdoba, Kevin Rico Gutiérrez, Silvia Marcela Ballesteros, José De la Hoz-Valle

**Affiliations:** 1 Subdirectorate of Clinical Performance Metrics, Fundación Santa Fe de Bogotá, Bogotá 110311, Colombia; 2 Burn Unit, Fundación Santa Fe de Bogotá, Bogotá 110311, Colombia; 3 Subdirectorate of Clinical Studies and Clinical Epidemiology, Fundación Santa Fe de Bogotá, Bogotá 110311, Colombia; kevin.rico@fsfb.org.co (K.R.G.);

**Keywords:** burn injury, anxiety, depression, patient-reported outcomes, psychological distress, burn rehabilitation, mental health, longitudinal follow-up

## Abstract

**Highlights:**

**What are the main findings?**

- Anxiety symptoms increased from hospitalization to three-month follow-up among adult burn patients.
- Severe burns were associated with higher odds of anxiety and depression symptoms at follow-up.

**What are the implications of the main findings?**

- Routine patient-reported mental health assessment should be integrated into burn care pathways.
- Early psychological support may be particularly important for patients with severe burn injuries.

**Abstract:**

Background: Burn injuries impose substantial physical and psychological burden beyond acute wound care. Anxiety and depressive symptoms are frequent but often underrecognized among burn survivors. Routine patient-reported mental health assessment during hospitalization and after discharge remains limited, particularly in Latin American burn care settings. This study described the early trajectory of patient-reported anxiety and depressive symptoms from hospitalization to three-month follow-up among adult burn patients treated at Fundación Santa Fe de Bogotá, Colombia. Methods: We conducted an observational cohort study including adult patients admitted to the Burn Unit between April 2022 and July 2023. Anxiety and depressive symptoms were assessed during hospitalization and at three months using the Zung Self-Rating Anxiety and Depression Scales. Scores ≥50 were considered clinically relevant. Results: A total of 118 patients with complete paired assessments were included. Clinically relevant anxiety symptoms increased from 19% during hospitalization to 29% at three months, while depressive symptoms were present in 32% and 29%, respectively. Severe burns and low educational attainment were associated with higher odds of clinically relevant symptoms at follow-up. Conclusions: Patient-reported anxiety and depressive symptoms were frequent during early burn recovery, supporting routine PROM-based mental health assessment and timely psychological follow-up.

## 1. Introduction

Anxiety and depression are recognized worldwide as frequent psychological sequelae among patients with burn injuries, largely due to the highly traumatic nature of these events [1,2,3,4]. Burn survivors often face acute pain, invasive procedures, functional limitations and uncertainty regarding their short-, medium- and long-term recovery. These factors can generate substantial psychological distress and interfere with overall rehabilitation [5,6,7,8].

In long-term follow-up studies, between 13% and 46% of patients present moderate to severe depressive symptoms from one to eight years after the injury [9]. In addition, in a cohort of 675 patients with non-mild burns, Chen et al. reported that approximately 40–47% presented anxiety and 39–42% depression after treatment, underscoring the high burden of post-burn psychological morbidity [10]. The presence of anxiety and depression adds substantially to the overall burden of burn injuries, highlighting the need for care models that address both the physical and psychological components of recovery [11].

Patient-Reported Outcome Measures (PROMs) provide a structured and patient-centered approach for evaluating these dimensions by capturing patients’ perceptions of their physical, emotional and social functioning after injury [12]. Beyond their descriptive role, PROMs offer a complementary modality for longitudinal follow-up by systematically documenting patients’ assessments of mood, functioning, and health-related quality of life. These instruments help detect symptoms of anxiety, depression, and distress that may remain unnoticed during routine consultations, support early identification, enable the monitoring of symptom trajectories over time, and enhance communication between patients and clinicians by incorporating patient priorities and experiences into care planning [13,14]. Evidence from stepped psychological care models suggests that screening is more clinically useful when results are shared with clinicians, discussed with patients, and linked to referral pathways rather than used as isolated assessments [15]. In burn care, where functional recovery, pain, body image concerns, and return to work evolve over time, PROMs can provide patient-centered information for value-based care by linking outcomes that matter to survivors with care coordination and quality improvement efforts [16].

Although PROMs are increasingly used in burn care, evidence on mental health trajectories after burn injuries remains limited. Some studies describe gradual psychological adaptation over time [17], while others show that patients may experience significant emotional challenges during the first year after hospitalization, with depressive and anxious symptoms that may originate in the acute phase and evolve during follow-up [6,7,8,18]. These inconsistencies reveal a gap in understanding the psychological outcomes of burn survivors when measured through these instruments.

The association between burn severity and psychological distress has been previously described. However, evidence from Latin American burn care settings remains scarce, particularly regarding the routine use of PROMs to monitor early emotional symptoms during the transition from hospitalization to outpatient recovery. Addressing this gap is essential for identifying prevention and intervention needs that may reduce the psychological burden of burn injuries and enhance person centered and value-based care. Therefore, this study aimed to describe the early trajectory of patient-reported anxiety and depressive symptoms from hospitalization to three-month follow-up among adult burn patients treated at Fundación Santa Fe de Bogotá, Colombia, and to explore routinely available clinical and sociodemographic factors associated with clinically relevant symptoms at follow-up.

## 2. Materials and Methods

### 2.1. Study Design

This observational cohort study included patients aged 18 years and older who were admitted to a specialized burn unit at Fundación Santa Fe de Bogotá between April 2022 and July 2023.

### 2.2. Context

The study was conducted at a high complexity institution in Bogotá, Colombia. Its specialized burn unit provides interdisciplinary care and comprehensive management for patients with burn injuries during both the acute phase and the outpatient follow-up period.

### 2.3. Participants

The study included patients if they were 18 years or older, were admitted to the Burn Unit between April 2022 and July 2023 with a diagnosis of chemical, thermal, electrical, radiation, contact, or friction burn, and had paired PROM data available for both the inpatient assessment and the three-month outpatient follow-up. Patients were excluded from the analysis if they declined the use of their clinical information for research purposes or if either the anxiety or depression assessment was missing at one of the two time points. All participants completed anxiety and depression assessments using the Zung Self-Rating Anxiety Scale (SAS) and the Zung Self-Rating Depression Scale (SDS) during hospitalization and again at the three-month outpatient follow-up [19,20].

The primary variables analyzed were patient reported symptoms of anxiety and depression, evaluated through the Zung scales. These instruments have been widely used in burn populations to identify the presence and severity of anxiety and depression symptoms [18,21], and have been validated in multiple regions, including the Colombian population [22,23].

Both scales consist of twenty items scored from 1 to 4 points. The total score was calculated by summing all items and multiplying the result by 1.25. Scores of 50 or higher indicated clinically relevant anxiety or depression. For this analysis, severe burn was used as an operational variable based on burn-center referral and specialized-care criteria routinely applied in the Burn Unit, informed by American Burn Association and European Burns Association frameworks [24,25]. This operational approach is consistent with burn literature describing severity as a function of burn size, depth, location, age, and underlying systemic disease [26]. In the present dataset, the variable incorporated available clinical information on burn depth, involvement of special anatomical areas, burn mechanism, and need for specialized burn-unit management. Because TBSA was not available as an individual analytic variable, it could not be incorporated into the analysis.

Clinically significant improvement was defined as a follow-up score of thirty nine or lower combined with a reduction of at least ten points from baseline [27]. The Zung Self-Rating Anxiety and Depression Scales were used as patient-reported symptom measures within the institution’s routine outcomes assessment pathway. These instruments were interpreted as screening tools for clinically relevant symptoms rather than diagnostic instruments. Although more contemporary tools such as PHQ-9, GAD-7, HADS, or PROMIS measures are increasingly used in current research and clinical practice, the Zung scales were selected because they were part of the institutional PROMs implementation process and had validated Spanish-language versions available for use in Colombia.

Sociodemographic variables, including sex and age, were collected for all participants. Clinical variables related to the type, degree and extent of the burn injury were also documented, as these characteristics may influence psychological outcomes such as anxiety and depression.

### 2.4. Data Collection

The institution has a dedicated outcomes unit responsible for designing and implementing strategies for measuring health outcomes across clinical, functional, and mental domains, including patient-reported outcome measures. The aim is to contribute to the generation of value in healthcare.

The principal investigator was a clinical psychologist affiliated with the Outcomes Unit who was trained in administering the SAS and SDS. These evaluations were conducted in person during the inpatient period and via telephone at the 3-month outpatient follow-up.

Completion of follow-up assessments was monitored through the institutional outcomes workflow. Through the collection, there was a risk of social desirability bias, where patients might choose responses on the scales that present themselves in a more favorable manner. For mitigating this bias, the assessments were conducted in confidential settings, and patients were informed before administering the scales that all responses were equally valid [28].

Because the primary analyses were based on within-patient comparisons between hospitalization and three-month follow-up, only patients with paired anxiety and depression assessments at both time points were included in the analytic cohort. Patients without complete paired PROM data were excluded from the main analysis, and no imputation was performed.

### 2.5. Sample Size

Sample size estimation was based on the study by Tang et al. [21], which reported mean changes of 3.06 points on the Self-Rating Anxiety Scale and 4.99 points on the Self-Rating Depression Scale over a three month follow-up. Assuming a two-tailed alpha of 0.05 and a statistical power of 80 percent to detect a similar within patient mean difference, the required sample size was estimated at 117 participants for anxiety symptoms and 70 participants for depression symptoms.

### 2.6. Outcomes

The scores from the SAS and SDS scales were analyzed as numerical variables. Scores above 49 were classified as clinically relevant anxiety or depressive symptoms. This cutoff was applied consistently at both the inpatient assessment and the three-month follow-up evaluation [27].

### 2.7. Statistical Analysis

A descriptive and inferential analysis was conducted to examine the relationship between sociodemographic and clinical variables and the SAS and SDS scores. Chi square tests were used to compare categorical variables when formal subgroup comparisons were conducted. For continuous or ordinal PROM scores stratified by sociodemographic and clinical characteristics, medians and interquartile ranges were reported descriptively; these descriptive patterns were not interpreted as formal chi-square results. Spearman correlation coefficients were calculated to assess the association between anxiety and depression scores during hospitalization and at follow-up. Correlation strength was interpreted according to Schober et al., defining values between 0.49 and 0.69 as moderate and between 0.70 and 0.89 as strong [29]. Paired tests were applied to evaluate within patient changes in anxiety and depression scores between the inpatient assessment and the three-month evaluation.

For the multivariable analysis, a logistic regression model was fitted using a Zung scale score of 50 or higher as the dependent variable for both anxiety and depression. Sociodemographic and clinical variables were included as independent predictors, and those showing statistical significance in the bivariate analysis were entered into the multivariate model. Logistic regression analyses were performed using Stata version 19, while all other statistical procedures were conducted using R version 4.5.1.

## 3. Results

### 3.1. Baseline Characteristics

**Figure 1:**
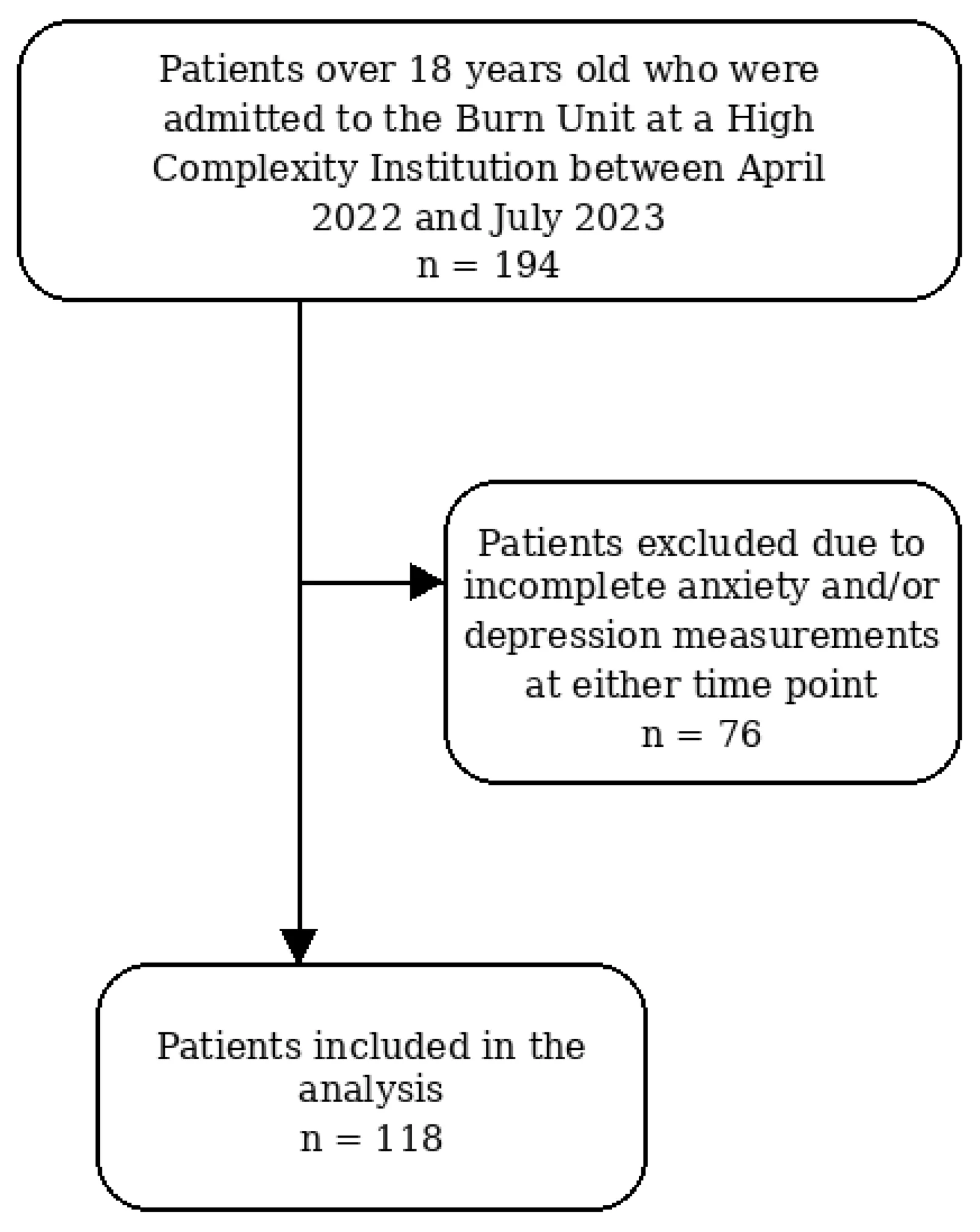
Flowchart of the patient inclusion process in the study.

A total of 194 patients were admitted to the Burn Unit between April 2022 and July 2023. Of these, 76 patients were excluded because complete paired anxiety and depression assessments at both time points were unavailable, leaving 118 patients who met the inclusion criteria and completed both the Zung Anxiety and Depression Scales during hospitalization and again at the three-month outpatient follow-up (Figure 1).

Most patients were female (70.34%). Second-degree burns were the most frequent injury (75.42%), followed by third-degree burns (24.58%). In addition, 14.41% of the sample was classified as having a severe burn. Detailed sociodemographic and clinical characteristics are presented in Table 1.

### 3.2. Prevalence and Distribution of Anxiety and Depression

The anxiety score was ≥50 in 19% of patients during hospitalization and increased to 29% at the three month follow-up. For depression, 32% of patients exceeded this threshold during the inpatient assessment and 29% did so at follow-up (Table 2). The overall median in-hospital anxiety score was 41 (IQR 36 to 48), decreasing slightly to 40 (IQR 35 to 50) at the three month evaluation. For depression, the median score during hospitalization was 43.5 (IQR 36 to 51), decreasing to 41 (IQR 31 to 51) at follow-up.

Descriptive stratified summaries suggested numerical differences between subgroups. Men had higher median anxiety and depression scores than women at both assessments, although these descriptive findings were not interpreted as definitive evidence of sex-related differences. Patients with severe burns had higher in-hospital anxiety scores (median 48 vs. 40) and depression scores (median 49 vs. 41) compared with those without severe burns, and these differences persisted at follow-up. A similar descriptive pattern was observed among patients with facial burns, who presented higher median values for both scales compared with those without facial involvement. No substantial differences were identified across burn mechanisms, graft requirement or injury location. These subgroup findings were considered exploratory and are provided to contextualize the regression analyses rather than as formal between-group comparisons.

Detailed anxiety and depression scores stratified by sociodemographic and clinical characteristics are provided in the Supplementary Materials (Tables S1 and S2).

### 3.3. Correlation Between Anxiety and Depression Scores

**Figure 2:**
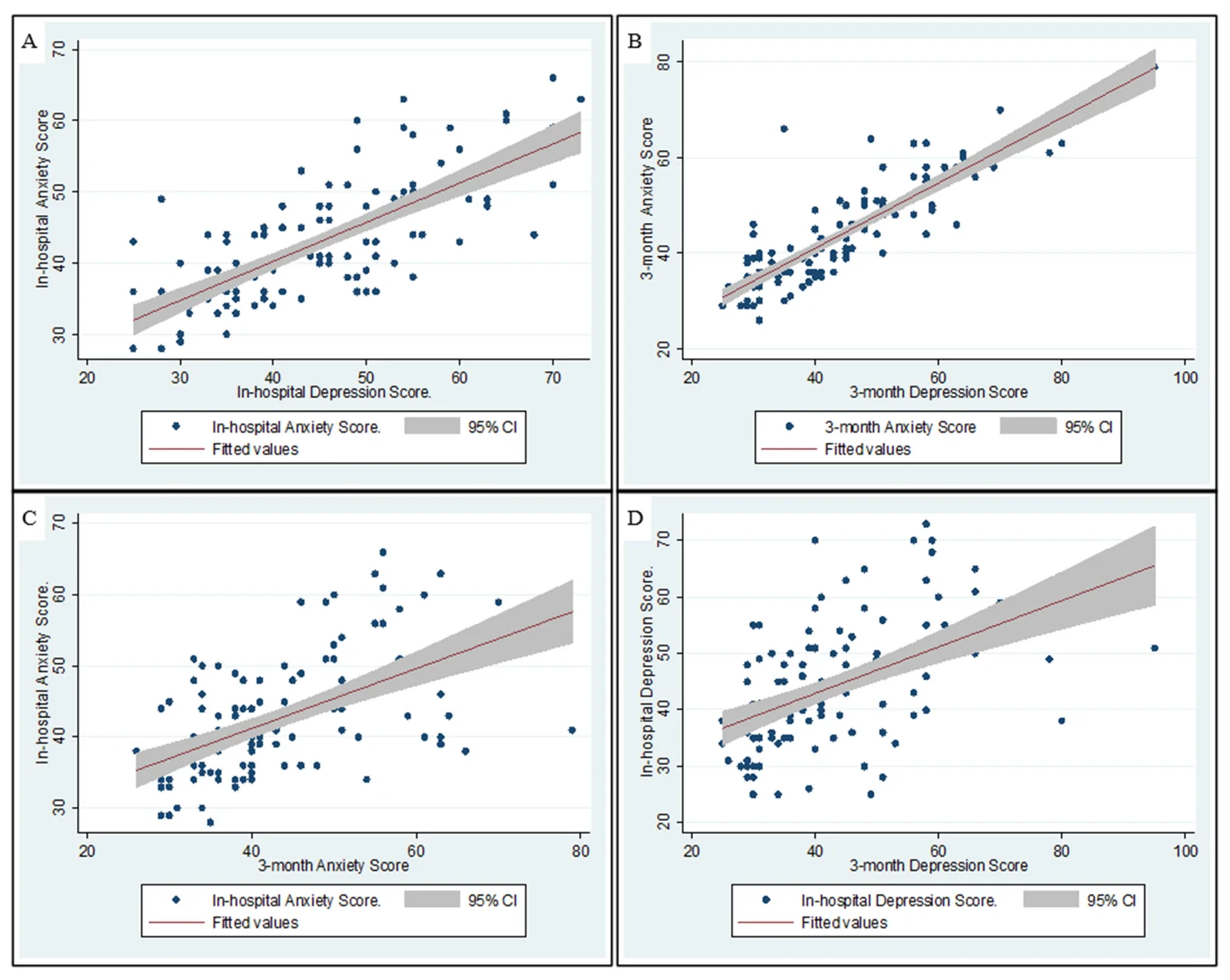
Correlation of anxiety and depression scores during hospitalization and at three-month follow-up in adult patients with burn injuries: (**A**) correlation between in-hospital depression and in-hospital anxiety scores; (**B**) correlation between three-month depression and three-month anxiety scores; (**C**) correlation between three-month and in-hospital anxiety scores; (**D**) correlation between three-month and in-hospital depression scores.

A strong positive correlation was observed between anxiety and depression scores during hospitalization, with a Spearman coefficient of 0.7177 (*p* < 0.0001). This association increased at the three month follow-up, where the correlation reached 0.8562 (*p* < 0.0001). When comparing scores across time, a moderate correlation was identified between inpatient and follow-up measurements for both anxiety (Spearman 0.5644, *p* < 0.0001) and depression (Spearman 0.5444, *p* < 0.0001). (Figure 2).

### 3.4. Logistic Regression Models

Multivariate logistic regression results are presented in Table 3 and Table 4. For anxiety at three months, low educational attainment was associated with higher odds of having a Zung anxiety score of 50 or higher (OR 3.43, 95% CI 1.29 to 10.5, *p* = 0.02). Patients classified as having a severe burn also showed higher odds of clinically relevant anxiety symptoms at follow-up (OR 6.24, 95% CI 1.69 to 26.6, *p* = 0.01) (Table 3).

For depression, the multivariable model indicated that severe burns were associated with higher odds of a Zung depression score ≥50 at three months (OR 3.36, 95% CI 1.11 to 10.5, *p* = 0.03). Low educational attainment was also associated with higher odds of depressive symptoms at follow-up (OR 2.74, 95% CI 1.06 to 7.85, *p* = 0.05). Female sex showed a non-significant trend toward higher odds of depression (OR 2.41, 95% CI 0.98 to 6.04, *p* = 0.06) (Table 4).

## 4. Discussion

This study describes the use of routine patient-reported outcome measures to monitor anxiety and depressive symptoms during hospitalization and at three-month follow-up among adults admitted to a specialized burn care unit at a high-complexity institution in Colombia. In this cohort, 19% of patients presented clinically significant anxiety and 32% presented clinically significant depression during hospitalization. At three months, the proportion with anxiety increased by 10%, whereas the prevalence of depression declined slightly. These trends were captured through the systematic use of patient-reported outcome measures, which allowed changes in emotional symptoms to be quantified rather than inferred. Despite minimal overall shifts in median Zung scale scores, the exploratory regression models based on routinely available clinical variables suggested relevant patterns associated with emotional outcomes. Lower educational attainment was associated with higher odds of clinically significant symptoms, while severe burns were associated with higher odds of anxiety and depression at follow-up. Taken together, these findings highlight the interplay between sociodemographic characteristics, injury severity and recovery context, underscoring the value of structured PROM-based assessment and the need for individualized and proactive psychological care in burn survivors.

Most participants were women, and the majority had second-degree burns, while approximately one in seven met criteria for a severe burn and nearly half of injuries occurred in the workplace. This contrasts with the report by Abarca et al. in Spain, where men accounted for around 60% of admissions and only 14% of burns were work-related [30]. In our cohort, this distribution suggests a different clinical and occupational profile, with greater representation of women and work-related injuries. Such context may be relevant when interpreting psychological outcomes, since occupational burns can involve return-to-work concerns, functional limitations, economic consequences, and rehabilitation needs that may influence anxiety and depressive symptoms during recovery.

Educational attainment varied considerably in our sample, where most patients had basic or secondary schooling and only a minority reported higher or professional education. In the regression models, low educational attainment was associated with higher odds of clinically significant anxiety and depressive symptoms at follow-up. This finding is consistent with those of Chlapecka et al., who conducted a cross-sectional study in more than 70,000 adults across Europe and reported that higher educational attainment was associated with lower odds of anxiety symptoms [31]. Together, these observations suggest that education may act as a contextual marker of access to information, coping resources, health literacy, and social support. In the burn recovery process, these factors may affect how patients understand wound care, rehabilitation recommendations, symptom progression, and indications for seeking additional clinical or psychological support.

Multivariable logistic regression provided additional insight into factors associated with clinically significant symptoms at three months. Sex-related differences should be interpreted cautiously. Although descriptive analyses suggested differences in anxiety and depression scores by sex, the multivariable model showed only a non-significant trend for depression. This differs from Masood et al., who reported greater psychological distress and lower resilience among women with burns [32]. The difference between these findings and previous studies may be related to the composition of the present cohort, including the predominance of women, the smaller number of men, and the proportion of work-related injuries. In addition, the variable sex did not capture gender-related factors such as caregiving roles, employment conditions, social support, body image concerns, or exposure to interpersonal violence. Patients with severe or facial burns also presented higher symptom levels, supporting the clinical relevance of injury severity and visible anatomical involvement when interpreting emotional burden. These findings are consistent with Palackic et al., who reported that even relatively common facial burns were associated with persistent deficits in physical and psychological health and significantly higher anxiety and depression scores [33], suggesting that psychological distress is common among burn survivors but is especially pronounced in those with facial involvement.

The longitudinal assessment of emotional symptoms showed only modest changes at the group level, with median anxiety and depression scores remaining relatively stable between hospitalization and the three-month follow-up. Despite these small shifts, the strong correlation between anxiety and depression at each time point and the moderate correlation of individual scores over time indicate that these conditions frequently co-occur and that early emotional responses tend to persist. Similar patterns have been reported by Panayi et al. and Kang-Auger et al., who documented elevated and enduring mental health risks among burn survivors well beyond the acute phase [34,35]. Within this context, PROM-based monitoring emerges as an essential tool for identifying patients whose symptoms do not improve and those who remain psychologically vulnerable despite clinical stabilization. In this sense, the present study contributes by documenting the feasibility and clinical usefulness of early longitudinal symptom monitoring in a Latin American burn care setting. In routine burn care, PROMs could be operationalized by administering symptom scales during hospitalization, repeating assessment after discharge, and using clinically relevant scores or worsening trajectories to prompt psychological evaluation, pain reassessment, rehabilitation support, or referral to mental health services. Taken together, these observations suggest that, in the absence of targeted intervention, early distress may evolve into longer-term psychological morbidity. This underscores the importance of integrating structured emotional assessment and timely psychological support into routine burn care and highlights the value of PROMs in detecting unmet psychological needs and guiding more targeted, patient-centered interventions.

The limitations of this study should be acknowledged. First, its single-institution design and follow-up limited to two time points may restrict generalizability and the characterization of longer-term psychological trajectories. Second, TBSA was not available as an individual variable for analysis, which limits comparability with previous burn studies and may have introduced residual misclassification of burn severity. Third, pre-existing psychiatric conditions, pain intensity, and psychosocial factors such as social support, employment conditions, and body image concerns were not systematically captured; therefore, residual confounding is likely, and these variables may have influenced the magnitude or direction of the observed associations. Fourth, patients without complete paired PROM assessments were excluded from the analytic cohort, which may have introduced selection bias if non-completers differed from included patients. Fifth, the Zung Self-Rating Anxiety and Depression Scales assess symptoms rather than clinical diagnoses and may be less comparable with more contemporary instruments. Finally, the sample size and number of events limited the complexity of the multivariable models, which should therefore be interpreted as exploratory. Future research with larger cohorts, standardized burn severity measures including TBSA, systematic assessment of psychiatric history, pain and psychosocial determinants, and extended follow-up is warranted.

## 5. Conclusions

Patient-reported anxiety and depressive symptoms were frequent among adult burn patients and persisted in a substantial proportion of patients during early recovery. In exploratory models based on routinely available clinical variables, severe burns and low educational attainment were associated with higher odds of clinically relevant symptoms at three-month follow-up. Routine patient-reported mental health assessment may help identify patients with persistent or emerging psychological needs and support earlier, more targeted psychological care in burn units.

## Figures and Tables

**Table 1:** Sociodemographic and Clinical Characteristics of Patients in a Specialized Burn Unit at Fundación Santa Fe de Bogotá (2022–2023).

Variable	*n*	%
**Sex**		
Female	83	70.34
Male	35	29.66
**Education**		
Primary basic education	11	9.40
Basic secondary school	68	58.12
Technologist	16	13.68
Professional	19	16.24
Postgraduate	3	2.56
No data	1	0.85
**Degree of burn**		
Grade 2	89	75.42
Grade 3	29	24.58
**Severe Burn**		
No	101	85.59
Yes	17	14.41
**Abbreviated Burn Severity Index (ABSI) mortality risk**		
<1%	24	22.43
1–10%	59	55.14
10–20%	16	14.95
30–50%	8	7.48
**Graft**		
No	70	59.32
Yes	48	40.68
**Burn agent**		
Non-thermal	27	25.23
Thermal	80	74.77
**Location**		
Workplace accident	56	47.46
Home	36	30.51
Other	26	22.03
**Special anatomical areas**		
No	9	7.63
Yes	109	92.37
**Face**		
No	70	59.32
Yes	48	40.68
**Assault**		
No	115	97.46
Heteroaggression	2	1.69
Self-harm	1	0.85

**Table 2:** Zung anxiety and depression scale score in patients admitted to the Burn Unit at Fundación Santa Fe de Bogotá (2022–2023) (*n* = 118).

Feature	*n* (%)
In-hospital Anxiety	
No	96 (81)
Yes	22 (19)
Anxiety at 3-months	
No	84 (71)
Yes	34 (29)
In-hospital depression	
No	80 (68)
Yes	38 (32)
Depression at 3-months	
No	84 (71)
Yes	34 (29)

**Table 3:** Logistic regression model for anxiety at 3 months greater than 49 points in patients admitted to the Burn Unit at a High-Complexity Institution.

Characteristics	Univariate			Multivariate		
	OR	95% CI	*p*-Value	OR	95% CI	*p*-Value
Low educational attainment	3.02	1.19, 8.78	0.03	3.43	1.29, 10.5	0.02
Severe Burn	2.56	0.88, 7.41	0.08	6.24	1.69, 26.6	0.01

**Table 4:** Logistic regression model for depression at 3 months greater than 49 points in patients admitted to the Burn Unit at a High Complexity Institution.

Characteristics	Univariate			Multivariate		
	OR	95% CI	*p*-Value	OR	95% CI	*p*-Value
Severe Burn	3.42	1.19, 10.0	0.02	3.36	1.11, 10.5	0.03
Low educational attainment	2.37	0.97, 6.48	0.07	2.74	1.06, 7.85	0.05
Sex	2.1	0.90, 4.89	0.08	2.41	0.98, 6.04	0.06

## Data Availability

The original contributions presented in this study are included in the article/Supplementary Materials. Further inquiries can be directed to the corresponding author.

## Supplementary Materials

The following supporting information can be downloaded at: https://www.mdpi.com/article/10.3390/ebj7030044/s1, Table S1. In-hospital anxiety scale scores and 3-months post-hospitalization according to sociodemographic and clinical variables in patients; Table S2. In-hospital depression scale scores and 3-months post-hospitalization according to sociodemographic and clinical variables in patients.

## Abbreviations

The following abbreviations are used in this manuscript:

PROMs	Patient-Reported Outcome Measures
SAS	Self-Rating Anxiety Scale
SDS	Self-Rating Depression Scale
ABSI	Abbreviated Burn Severity Index
OR	Odds Ratio
CI	Confidence Interval
IQR	Interquartile Range
TBSA	Total Body Surface Area
ABA	American Burn Association
EBA	European Burns Association
