# Supplementary material for: Anxiety and Depression Symptoms in Patients with Burn Injuries at Fundación Santa Fe de Bogotá, Colombia: Study of Patient-Reported Outcomes
Source: Eur Burn J. 2026 Aug 13;7(3):44. doi: 10.3390/ebj7030044 (PMC13511026; doi:10.3390/ebj7030044)
Supplement: Supplementary file 1 [file ebj-07-00044-s001.zip › ebj-4308302-supplementary.pdf]

Supplementary material

Table S1. In-hospital anxiety scale scores and 3-months post-hospitalization according to sociodemographic and clinical variables in patients.

	**In-Hospital Anxiety Score**					**Anxiety Score 3 Months**				
	n	Median	P25	P75	P-Value	n	Median	P25	P75	p-value
Sex										
Female	83	40	36	48	0.007	83	40	34	48	0.057
Male	35	44	40	50		35	46	36	51	
Total	118	41	36	48		118	40	35	50	
Schooling										
Primary Basic Education	11	39	36	41	0.244	11	40	36	48	0.175
Basic Secondary School	68	42	36	49		68	42	35	55.5	
Technologist	16	46.5	37.5	50.5		16	42.5	38	49	
Professional	19	40	34	44		19	35	34	43	
Postgraduate	3	43	36	45		3	43	34	44	
Total	117	41	36	48		117	40	35	50	
The Degree of The Burn										
Grade 2	89	41	36	49	0.257	89	41	36	50	0.204
Grade 3	29	40	36	48		29	39	34	48	
Total	118	41	36	48		118	40	35	50	
Severe Burn										
No	101	40	36	46	0.038	101	40	35	50	0.269
Yes	17	48	40	51		17	44	39	56	
Total	118	41	36	48		118	40	35	50	
ABSI Mortality Risk										
<1%	24	44	36	49	0.211	24	41	35	51	0.176
1-10%	59	39	35	48		59	40	34	50	
10-20%	16	42	39.5	50		16	47.5	40.5	57	
30-50%	8	44.5	40.5	49		8	39	36.5	40	
Total	107	41	36	49		107	40	35	51	
Graft										
No	70	41	36	48	0.762	70	41	34	54	0.579
Yes	48	40.5	36	48.5		48	40	36	48.5	
Total	118	41	36	48		118	40	35	50	
Burn Agent										
Non-Thermal	27	40	36	48	0.457	27	40	36	51	0.971
Thermal	80	41	36	49		80	41	35	50.5	
Total	107	41	36	49		107	41	35	51	
Location										
Workplace Accident	56	40	36	47		56	41	34	55	
Home	36	42	36	49		36	39	35.5	49	
Other	26	41	36	50		26	40.5	34	50	
Total	118	41	36	48		118	40	35	50	
Special Anatomic Areas										
No	9	43	34	45	0.327	9	38	30	45	0.165
Yes	109	41	36	49		109	40	35	51	
Total	118	41	36	48		118	40	35	50	
Face										

No	70	39.5	35	45	0.011	70	40	34	49	0.097
Yes	48	43	39	50		48	43	36	54.5	
Total	118	41	36	48		118	40	35	50	
Assault										
No	115	41	36	48	0.610	115	40	35	51	0.739
Heteroaggression	2	47.5	36	59		2	37.5	29	46	
Self-Harm	1	36	36	36		1	40	40	40	
Total	118	41	36	48		118	40	35	50	

Table S2. In-hospital depression scale scores and 3-months post-hospitalization according to sociodemographic and clinical variables in patients.

	**In-Hospital Depression Score**					**Depression Score 3-Months**				
	n	Median	P25	p75	p-value	n	Median	P25	p75	p-value
Sex										
Female	83	40	35	50	0.001	83	39	31	49	0.014
Male	35	49	41	55		35	45	39	59	
Total	118	43.5	36	51		118	41	31	51	
Schooling										
Primary basic education	11	39	34	48	0.191	11	45	36	53	0.078
Basic secondary school	68	44	35	54		68	41	31	58	
Technologist	16	48	40.5	58.5		16	43	40	52	
Professional	19	39	34	48		19	31	29	44	
Postgraduate	3	45	39	49		3	41	29	50	
Total	117	43	36	51		117	41	31	51	
The Degree of The Burn										
Grade 2	89	45	36	53	0.299	89	43	33	56	0.233
Grade 3	29	41	34	51		29	38	38	50	
Total	118	43.5	36	51		118	41	31	51	
Severe Burn										
No	101	41	35	51	0.016	101	40	31	49	0.042
Yes	17	49	46	51		17	50	38	58	
Total	118	43.5	36	51		118	41	31	51	
ABSI Mortality Risk										
<1%	24	45.5	37	56.5	0.212	24	45	33	51	0.675
1-10%	59	40	34	51		59	40	31	53	
10-20%	16	48.5	40	54.5		16	45.5	35	57	
30-50%	8	46	44	49.5		8	38.5	34.5	48	
Total	107	45	35	53		107	41	31	51	
Graft										
No	70	43	36	51	0.967	70	41	31	56	0.873
Yes	48	44.5	35	53.5		48	39.5	31	50.5	
Total	118	43.5	36	51		118	41	31	51	
Burn Agent										
Non-thermal	27	40	35	50	0.206	27	41	31	51	0.513
Thermal	80	45	36	53		80	41	32	56	
Total	107	44	36	53		107	41	31	51	
Location										
Workplace accident	56	40	35	50		56	41	31	56	
Home	36	46	38.5	53.5		36	40	32	50.5	
Other	26	44.5	36	54		26	44.5	30	50	
Total	118	43.5	36	51		118	41	31	51	
Special Anatomic Areas										
No	9	41	35	50	0.499	9	30	30	40	0.030
Yes	109	45	36	53		109	41	33	51	
Total	118	43.5	36	51		118	41	31	51	
Face										
No	70	40.5	34	51	0.053	70	39.5	31	51	0.096
Yes	48	45.5	39	53.5		48	44	35	56	

Total	118	43.5	36	51		118	41	31	51	
Assault										
No	115	43	35	51	0.701	115	41	31	51	0.658
Heteroaggression	2	45	36	54		2	36.5	29	44	
Self-harm	1	51	51	51		1	45	45	45	
Total	118	43.5	36	51		118	41	31	51	
